# Influence of head orientation and implantation site of a novel transcutaneous bone conduction implant on MRI metal artifact reduction sequence

**DOI:** 10.1007/s00405-022-07272-3

**Published:** 2022-01-24

**Authors:** Emile Talon, Wilhelm Wimmer, Arsany Hakim, Claus Kiefer, Manuela Pastore-Wapp, Lukas Anschuetz, Georgios Mantokoudis, Marco D. Caversaccio, Franca Wagner

**Affiliations:** 1 Department of Otorhinolaryngology, Head and Neck Surgery, Inselspital, Bern University Hospital, and University of Bern, Bern, Switzerland; 2 Hearing Research Laboratory, ARTORG Center for Biomedical Engineering Research, University of Bern, Bern, Switzerland; 3 Department of Diagnostic and Interventional Neuroradiology, Inselspital, Bern University Hospital, and University of Bern, Bern, Switzerland

**Keywords:** BONEBRIDGE, Artifact reduction, Magnetic resonance imaging, WARP, SEMAC-VAT WARP

## Abstract

**Purpose:**

The use of magnetic resonance imaging (MRI) is often limited in patients with auditory implants because of the presence of metallic components and magnets. The aim of this study was to evaluate the clinical usefulness of a customized MRI sequence for metal artifact suppression in patients with BONEBRIDGETM BCI 602 implants (MED-EL, Innsbruck, Austria), the successor of the BCI 601 model.

**Methods:**

Using our in-house developed and customized metal artifact reduction sequence (SEMAC-VAT WARP), MRI artifacts were evaluated qualitatively and quantitatively. MRI sequences were performed with and without artifact reduction on two whole head specimens with and without the BCI 602 implant. In addition, the influence of two different implantation sites (mastoid versus retrosigmoid) and head orientation on artifact presence was investigated.

**Results:**

Artifact volume was reduced by more than the 50%. Results were comparable with those obtained with the BCI 601, showing no significant differences in the dimensions of artifacts caused by the implant.

**Conclusion:**

SEMAC-VAT WARP was once more proved to be efficient at reducing metal artifacts on MR images. The dimensions of artifacts associated with the BCI 602 are not smaller than those caused by the BCI 601.

## Introduction

Magnetic resonance imaging (MRI) is widely used for clinical evaluation. The presence of metal components in the region being analyzed may, however, cause safety concerns or produce artifacts on the image, which may limit diagnostic utility [1]. Metal components are present in hearing prostheses and in cochlear, middle ear, and bone conduction implants [2]. Candidates for hearing implants are typically subjected to preoperative and postoperative follow-up imaging, usually either computed tomography (CT) or MRI scans. These imaging techniques can be exploited to provide new tools for planning bone conduction hearing implant surgery [3] or for analysis of temporal bone [4] or clinical follow-up. For this reason, MRI compatibility is an important requirement for hearing implants [5, 6]. MRI compatibility is mainly considered from the point of view of safety; nevertheless, the diagnostic value of MRI scans acquired in patients with an implant must also be ensured. The shape, dimensions, and intensity of imaging artifacts depends on multiple factors, such as the implant geometry and construction materials, the position of the head within the MRI scanner, location of the implant on the temporal bone, the MRI scanner model used, the acquisition protocol, and the inclusion or exclusion of magnet reduction sequences (MARS) [7].

Previously conducted studies have analyzed the quality of MRI with MRI-compatible cochlear implants [8, 9], while the impact of our in-house primarily self-built sequence for metal artefact suppression (SEMAC-VAT WARP) was analyzed on two head specimens implanted with the bone conduction hearing implant BONEBRIDGE™ BCI 601 (BB; MED-EL, Innsbruck, Austria) [10]. Our hereinafter by Siemens customized SEMAC-VAT WARP sequence enabled acquisition of images of higher diagnostic value compared to MRI sequences obtained without it.

**Fig. 1:**
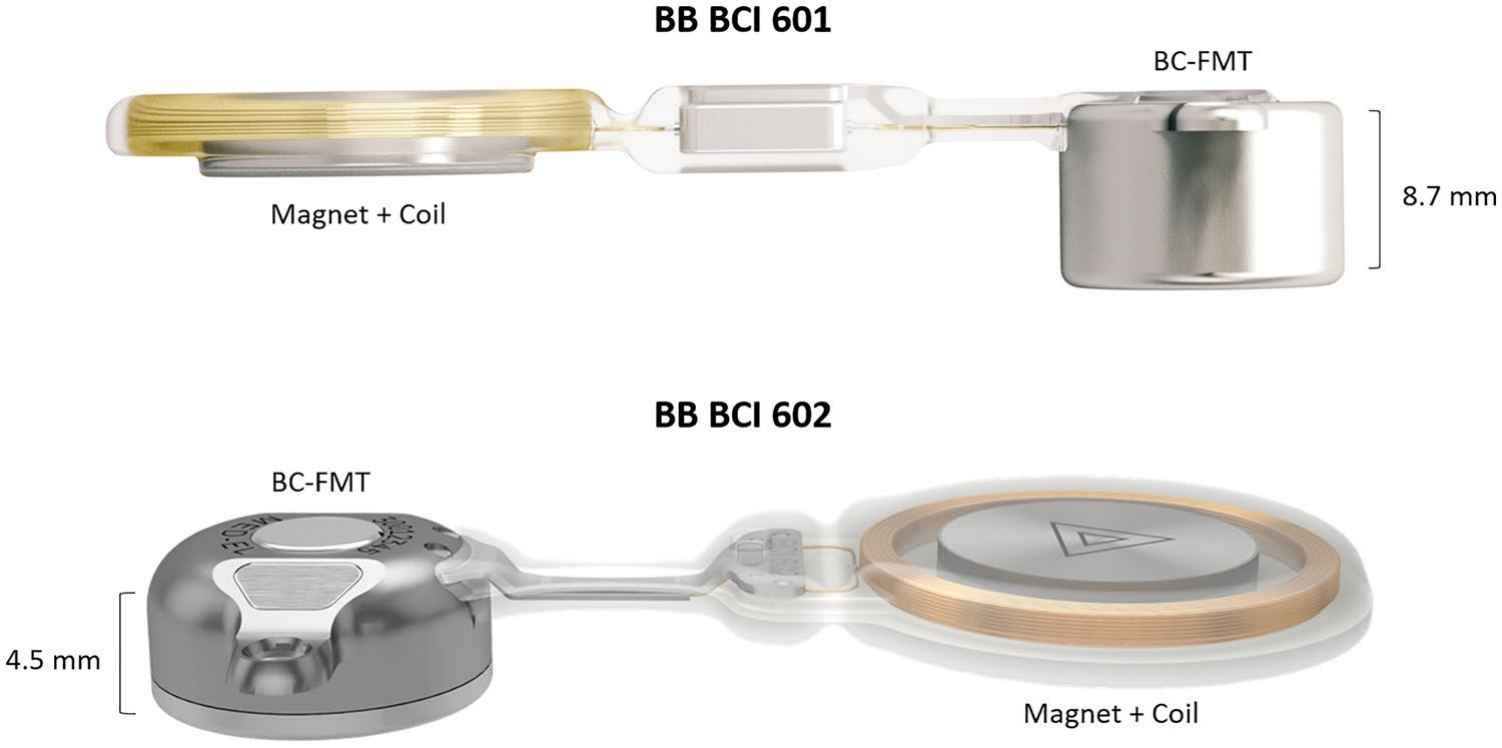
BONEBRIDGE™ BCI 601 (top) and BCI 602 (bottom) (MED-EL, Innsbruck, Austria) implants with the required dimensions for the bone bed to accommodate the BC FMT transducers. Images courtesy of MED-EL

The BB is indicated for conductive or mixed hearing loss and single-sided deafness [11, 12]. The BCI 601 implant was introduced in 2012, while its successor, the BCI 602, was released in 2019. Both devices consist of an externally worn audio processor and an implantable part. The most significant element of the implant is the transducer, which generates the mechanical vibration required to stimulate the inner ear. In both devices the transducer used is a bone conduction floating mass transducer (BC-FMT). The main difference between the older BCI 601 and the newer BCI 602 is the depth of bone bed needed for the BC-FMT, which was reduced from 8.7 to 4.5 mm, as shown in Fig. 1.

Although the BCI 601 and the BCI 602 are both safe up to 1.5 T field strength, the metallic and magnetic components of the devices cause artifacts in the MR images obtained [10, 13]. Various metal artifact reduction sequences have been developed in recent years, such as the slice encoding for metal artifact correction (SEMAC) [14, 15], view angle tilting (VAT) [14, 16], hybrid versions (SEMAC-VAT) [14, 17] and a combination of VAT with fast spin-echo sequences with high bandwidth (WARP, Siemens Healthcare, Erlangen, Germany). The main objective of the present study was to evaluate the diagnostic usefulness of our in-house developed and customized sequence for MRI metal artifact reduction (SEMAC-VAT WARP) [10] with the recently introduced BB BCI 602 [18] for MR images obtained with a 1.5 T scanner. The secondary objectives were to investigate whether the implant position and head position affect MRI artifacts.

## Materials and methods

### Study design and preparation

**Fig. 2:**
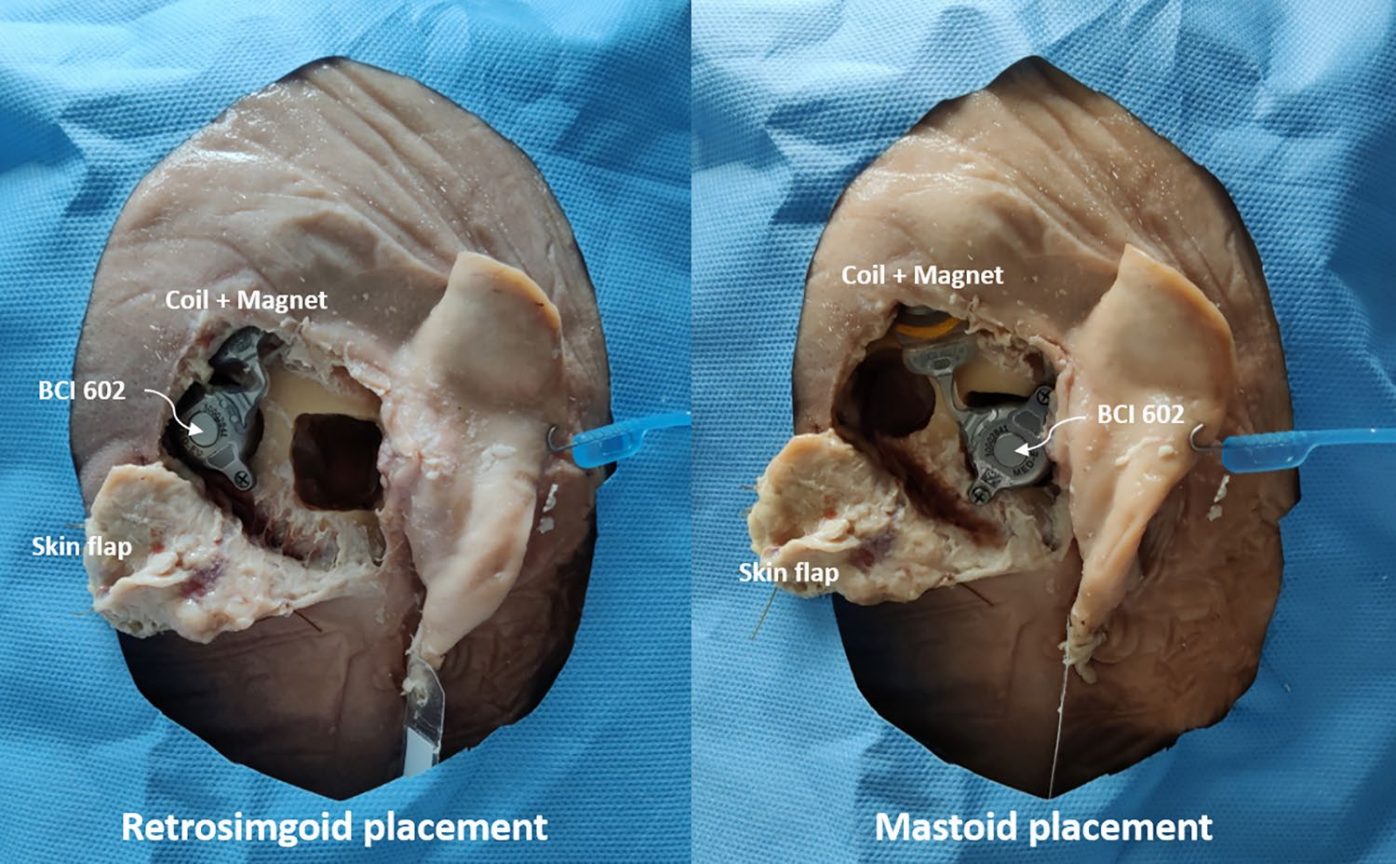
Retrosigmoid and mastoid implant positions used in the study

The methods applied in this study are based on those used in studies of the BB BCI 601 model [10]. To ensure comparability of the results, the same protocol was followed in the present study. Two Thiel-fixed whole human heads were [19] implanted with BB BCI 602 implants on the right ear side. Before implantation, high resolution CT and native MRI scans were obtained for both specimens. The BB was implanted in a mastoid and retrosigmoid location in each specimen, as indicated in Fig. 2. The implantation was performed according to the standard procedure involving a mastoidectomy. The receiver coil of the implant was placed in such a way as to enable realistic audio processor positioning. The implant was then immobilized with two self-drilling screws. The study was approved by the local institutional review board (KEK-BE 2016-00887).

### Image acquisition

All imaging was performed using a 1.5-T MRI scanner (MAGNETOM Avantofit; Siemens Medical Solution, Erlangen, Germany) with a 12-channel head coil [10]. The pre-implantation MRI protocol included a T1-weighted (T1w) sagittal sequence, a coronal T2-weighted (T2w), and an axial reconstruction. The post-implantation MRI protocol was applied identically to both head specimens. First, without artifact reduction, the following sequences were performed:

A T1w (slice thickness [ST] 4 mm) and a T2w (ST 4 mm) over the whole brain, a coronal T2w (ST 2 mm) over the temporal bone, an axial T1w (ST 2 mm) over the temporal bone, and a constructive interference in steady state (ST 0.6 mm). Second, the SEMAC-VAT WARP (echo trains per slice: 7, VAT: 100%, SEMAC: 20, turbo factor: 15) artifact reduction sequence was applied to axial T1w and coronal T2w sequences. Images of the head were also acquired with the head in two different positions. The first scans were carried out with the head positioned in the middle of the head shell, fixed on the right and left by 2 head cushions corresponding to the standard patient position for MR scans. For the second scans, the cadaver heads were positioned with the head turned by 30° to the implanted side. In our experience, this angle is comfortable enough for patients to avoid generating movement artifacts for the duration of the MR scan. All the image acquisition parameters are the same as described in the previously conducted study [10].

### Image analysis

Two experienced neuroradiologists (F.W. and A.H.), who also analyzed the images in the previous study [10], independently evaluated the MRI scans. Afterwards, a consensus reading was performed by both neuroradiologists. The same brain structures were graded on the ipsilateral and the contralateral side of the implant with and without SEMAC-VAT WARP for the T1w and the T2w sequences: the frontal lobe, parietal lobe, temporal lobe, occipital lobe, internal auditory canal, and the petrous bone. The brainstem and skull base were evaluated without separation of the sides. The image quality definition followed the grading system introduced by Wagner et al. [8], ranging from 0 to 3, corresponding to a completely deficient, insufficient, good, or the best possible image quality, respectively. The window values (picture contrast and brightness) were individually adjusted by the examiners. Measurements of total and artifact-affected volumes for all sequences were performed through manual segmentation on a 3D Slicer [20]. The 3D Slicer was also used to perform an anatomical landmark-based volume registration of the CT scans with the MR sequences, used mainly for visualization purposes. The percentages of the artifact volume compared to the total head volumes were measured by manual segmentation. The volume percentages were obtained from T1w turbo spin echo (TSE) axial images with a slice thickness of 3 mm in accordance with the method of Sharon et al. [21]. Voxels were considered part of an artifact if the visibility of the structures in the MRI scan was completely or highly compromised (values 0 and 1 on the scale reported by Wagner et al. [8]).

### Statistical analysis

Statistical analysis was used to determine the difference between the MR images acquired with and without the SEMAC-VAT WARP in comparison to the pre-implant whole head specimen scans. Furthermore, the difference in image quality between the two different implant locations was evaluated. The statistical analyses were performed using IBM SPSS Statistics for Windows Version 25.0. All statistical tests were 2-sided; a *p* value < 0.05 was considered as statistically significant. The inter-rater reliability was calculated according to Cohen’s kappa. Non-parametric Wilcoxon signed-rank tests were used to compare the ratings of the data sets with and without artifact reduction.

## Results

**Fig. 3:**
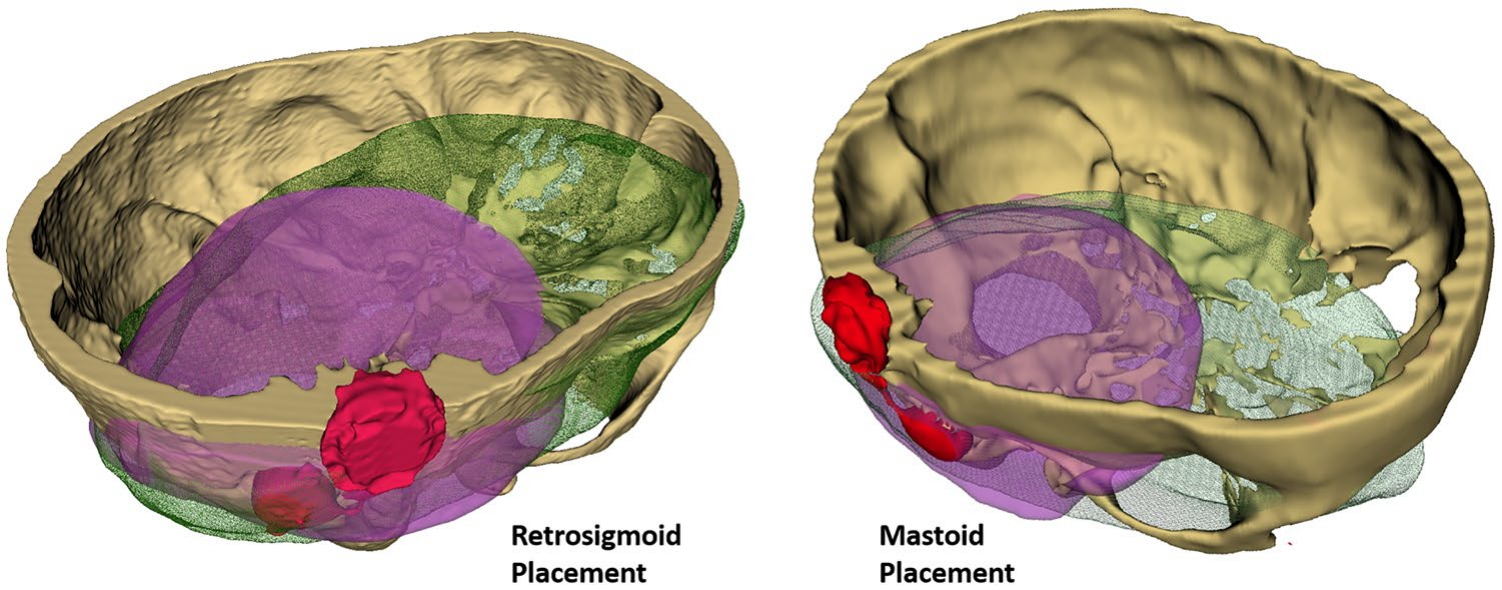
Three-dimensional visualization of the artifact volumes reported in Table 1 for implants in Head 1 in a retrosigmoid (left figure) and mastoid (right figure) position. Green-shaded areas show the artifact extent without the artifact reduction sequence, while purple shading indicates the artifact extent when the SEMAC-VAT WARP sequence is applied

**Fig. 4:**
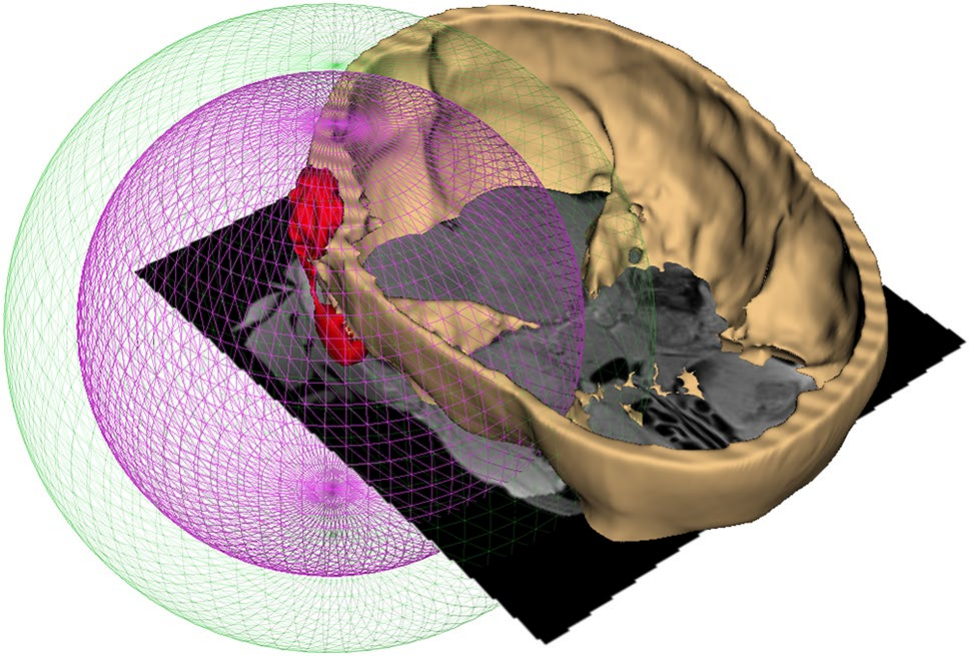
Three-dimensional visualization of simplified artifact dimensions on the registered MRI-CT images. The green sphere represents the artifact extent without the artifact reduction sequence (radius = 9 cm). The purple sphere shows the artifact extent when the SEMAC-VAT WARP sequence is applied (radius = 7 cm). The spheres are centered around the implant (shown in red) placed in the mastoid position

**Fig. 5:**
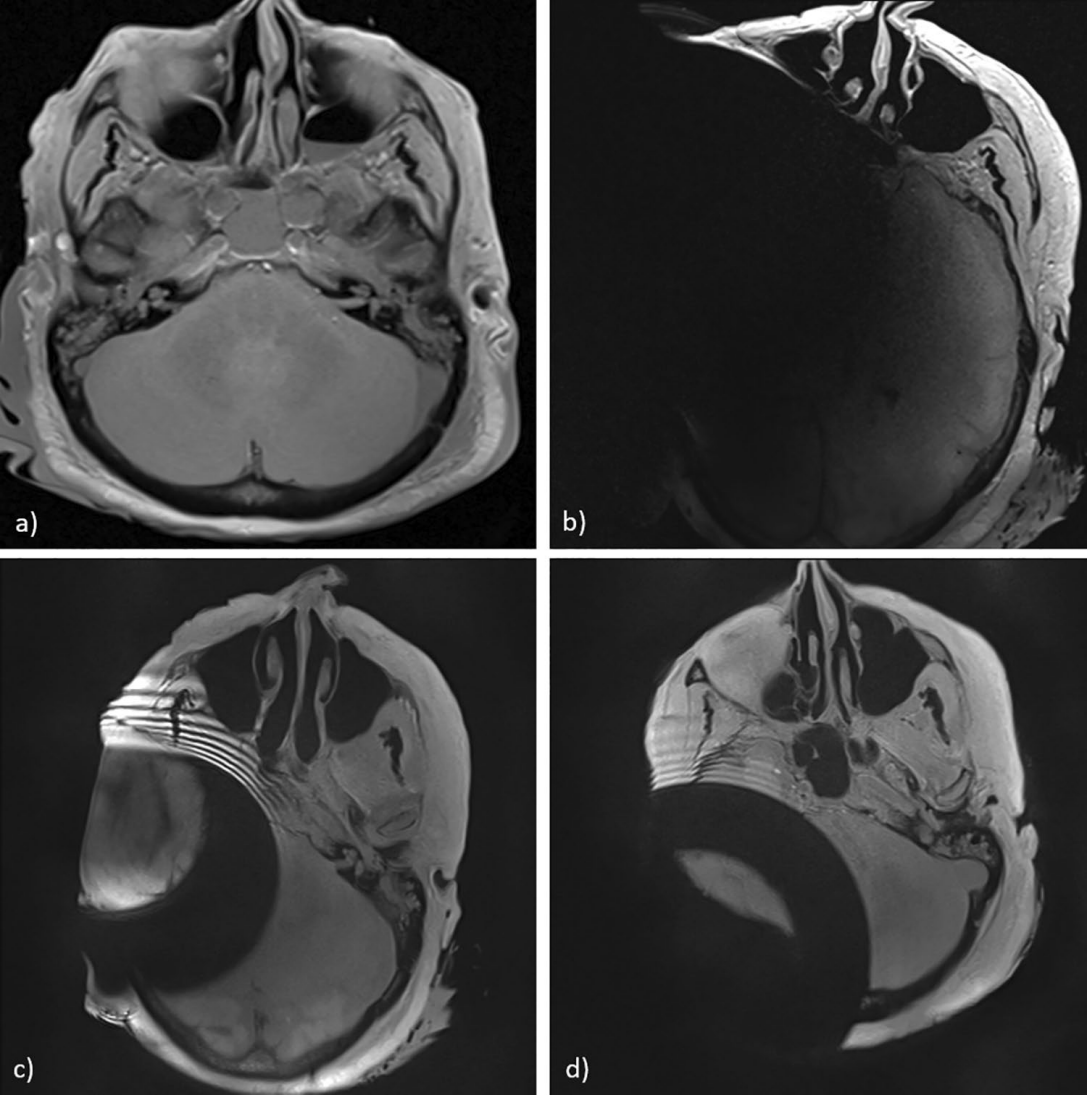
MR images from one of the two head samples. All MR images shown here are taken from an axial T1 sequence. **a** Pre-implantation scan used as a reference. **b** Post-implantation scan without metal artifact reduction sequences. **c** Post-implantation scan with SEMAC-VAT WARP artifact reduction sequence: implant placed on the mastoid. **d** Post-implantation scan with SEMAC-VAT WARP artifact reduction sequence: implant placed in retrosigmoid location

The inter-rater reliability was confirmed through Cohen’s kappa = 0.602, *p* < 0.001. The artifacts were clearly detectable on post-implantation MRI sequences. However, the results showed a substantial decrease in the dimensions of the artifacts following application of the SEMAC-VAT WARP sequence compared to MRI without artifact reduction. Postoperative MRI scans without the artifact reduction sequence showed large artifacts with a diameter up to 9 cm (SD ± 2.5 cm) from the implantation site. The artifact roughly resembles a spherical black shadow (see Fig. 5b). Without the artifact reduction algorithm, the artifact volume was 55.9% (SD ± 0.9) of the total volume. When the SEMAC-VAT WARP was applied, the proportion dropped to 26.4% (SD ± 0.6) in the case of an implant in the retrosigmoid position and 28.5% (SD ± 1.7) in the case of mastoid placement. The volume measurements are reported in Table 1, visualized in Fig. 3 for comparison purposes, and modeled as spheres in Fig. 4.

**Table 1:** Percentages of artifact volumes with and without artifact reduction with respect to the total MRI volume for the two specimens and implant locations

	Without artifact reduction (%)	With artifact reduction: retrosigmoid location (%)	With artifact reduction: mastoid location (%)
Head 1	56*.*9	27*.*1	30*.*2
Head 2	55*.*0	25*.*8	26*.*8

The artifact reduction sequence improved the visibility of the different regions, especially on the implanted side of the head (Fig. 5). Major improvements were observed for the parietal, frontal, temporal, and occipital lobes. In the post-implantation MRI, the artifacts were very distinct and prevented any evaluation of anatomical structures, whereas when the SEMAC-VAT WARP sequences were applied, only moderate artifacts were present, enabling the assessment of central brain structures. However, regions close to the implant remained obscured by metal artifacts despite the artifact reduction sequence. These regions included the cerebellum, the internal auditory canal, and the petrous bone. The artifact in the scans with SEMAC-VAT WARP sequences mainly consisted of a black ring superimposed on the image, as shown in Fig. 5c and d, together with rings of artifact fluctuations next to the black ring. The radius of the artifact region was 6.5 cm (SD ± 0.2) when the implant was in the mastoid position and 7 cm (SD ± 0.3) when it was in the retro-sigmoidal position.

Perfect visibility of all anatomical structures on the contralateral side (non-implanted side) was obtained when metal artifact reduction was applied. Without reduction, the major structures were still identifiable, but there were minor artifacts on internal canal, petrous bone, and cerebellum.

For the brainstem and skull base, the SEMAC-VAT WARP sequence only slightly improved the MRI quality. The difference in image quality with and without SEMAC-VAT WARP was statistically significant (see Table 2).

**Table 2:** Differences in diagnostic usefulness between MRI scans with and without SEMAC-VAT WARP in the presence of the BB BCI 602 implant

	Implant position	*Z* index	*p *value	Significance
Ipsilateral side	Retrosigmoid	− 3.21	0.001	***
	Mastoid	− 3.02	0.002	***
Contralateral side	Retrosigmoid	− 2.92	0.004	***
	Mastoid	− 2.60	0.009	***

Ipsi- and contralateral sides are compared, together with the two possible implant locations

***Statistically significant difference

No statistically significant differences were found in the diagnostic value of the MRI between the two implant locations for both comparisons on the implanted side of the head (ipsilateral) and the contralateral side. The artifact volume percentages reported in Table 1 are similar for the retrosigmoid and mastoid implant location (26.42% versus 28.48%), suggesting once again that there is no significant difference between implant locations. The anatomical regions that could not be conclusively assessed despite application of the SEMAC-VAT-WARP sequence were on the implanted side in the internal auditory canal, the cerebellum, and the petrous bone as well as the anatomical structures of the brainstem and central skull base, which were not separated according to side.

Moreover, the Wilcoxon signed-rank test showed no statistically significant influence of the head position during the MR image acquisition on the quality of the image obtained.

## Discussion

The SEMAC-VAT WARP sequence considerably increases the clinical usefulness of MRI sequences when applied in patients who have the bone conduction implant BONEBRIDGE™ BCI 602. Application of the metal artifact reduction sequence to the images greatly improved the image quality on the implanted side due to a reduction of the size of the artifact. The non-implanted side had the same image quality as the pre-implantation MR reference scans. In addition, the results shown in Table 1 underline the usefulness of the metal artifact reduction sequence for reducing artifact volumes.

The fact that the BCI 602 requires a shallower implant bed than the BCI 601 might suggest that metal artifacts on MR images would be reduced. However, the results obtained in the present study do not back up this hypothesis. Metal artifact dimensions on MRI without SEMAC-VAT WARP sequence are comparable to the ones obtained in the previous study on the BCI 601 [10] (9 ± 2.5 cm for the BCI 602 versus 10 ± 2 cm for the BCI 601). The impact of the SEMAC-VAT WARP on axial T1w images is similar for both implants. The Z index for the implanted side was − 2.85 for the BCI 601 [10] and − 3.21 or − 3.02 for the BCI 602 (depending on implant location, see Table 2).

The lateral head inclination during image acquisition did not influence the area or the position of the artifact. However, some other studies on cochlear implants have analyzed the influence of anteflexion and retroflexion during imaging. Hyperextension of the cervical spine caused a displacement of the artifact region toward the inner ear [22, 23] Anterior and posterior head tilting were, however, excluded from our analysis, because they are considered uncomfortable for the patient and, therefore, not clinically realistic. Even if some improvements might be possible with head tilting, they remain marginal and not readily repeatable with respect to the action of artifact reduction sequences [23].

In this study, cadaver specimens were preferred over performing measurements on live patients. This decision was made to enable a direct comparison with the previous study on the BCI 601 [10]. The use of Thiel-fixated head specimens was also proven to represent a suitable model for these kind of measurements [10, 19]. The choice of performing measurements on cadaver head could, moreover, allow a quicker and easier ethical approval process.

The methods used in the study could be directly applied on patients implanted with the BB BCI 602. We do not expect substantial differences in artifact size, as we compared the artifact suppression also in a patient [10]. Postoperative MRI scans represent the best option for follow-up clinical analysis: we believe, therefore, that easily repeatable artifact reduction techniques such as the SEMAC-VAT WARP will support increased use of MRI for patients with hearing implants.

The impact of a metal artifact reduction sequence after BB BCI 602 implantation was also analyzed by Utrilla et al. [13]. In their study, MRI was performed with a Signa® 1.5 T scanner (General Electrics Healthcare, Medical Systems). These authors used a different brand-specific metal artifact reduction method (MAVRICK). Moreover, the influence of the less widespread middle fossa approach for surgical placement of the BB on the MRI quality was evaluated. The results obtained by Utrilla et al. [13] when the implant was placed on the mastoid are similar to those obtained in the present study. Artifact size after application of the metal artifact reduction sequences is comparable (6.3 cm versus 7.0 cm). However, Utrilla et al. showed that the middle fossa approach led to much smaller artifacts (3.4 cm). Utrilla et al. [13] did not directly compare the two devices BB BCI 601 and 602 in the standard implant locations of retrosigmoid and mastoid: BCI 601 was implanted in the mastoid location and the BCI 602 in the retrosigmoid location. However, in both cases, the artifacts had a radius of approximately 6.3 cm when the MAVRICK sequence was applied. These results are in agreement with ours, which showed no statistically significant differences in artifact dimensions between retrosigmoid and mastoid implant location. Qualitative and quantitative results reported in this study and from previous work [10, 13] suggest that the metal artifact reduction sequence SEMAC-VAT WARP generally improves image quality. However, contrary to the observations by Utrilla et al. [13], we did not find smaller metal artifacts with the newer BCI 602 implant than with the older model.

Some limitations of our study should be noted. First, the MRI sequence is proprietary needs to be purchased. No direct artifact volume comparison was made, since the T1 axial sequences were not performed on the whole head but on slices of the temporal bone with different thicknesses. Moreover, more specimens need to be imaged to confirm our findings. Applying our measurements to patients wearing an external headband with the BCI 602 could also represent a first step toward in-vivo artifact volume measurements validation. The impact of the less common middle fossa implant location should also be analyzed with our image acquisition method to confirm its influence on the dimensions of the MRI metal artifact.

## Conclusions

MRI at 1.5 T is considered safe for patients with BB BCI 602. Metal artifact reduction sequences have been shown to significantly improve MR image quality, increasing clinical relevance, especially on the implanted side of the head. The efficiency of SEMAC-VAT WARP has been demonstrated in previous work and further confirmed in this study. However, the BCI 602 implant was not shown to produce smaller artifacts than the BCI 601 implant, despite the transducer being smaller than its predecessor.
